# Indoor 3D Reconstruction of Buildings via Azure Kinect RGB-D Camera

**DOI:** 10.3390/s22239222

**Published:** 2022-11-27

**Authors:** Chaimaa Delasse, Hamza Lafkiri, Rafika Hajji, Ishraq Rached, Tania Landes

**Affiliations:** 1 College of Geomatic Sciences and Surveying Engineering, Institute of Agronomy and Veterinary Medicine, Rabat 6202, Morocco; 2 ICube Laboratory UMR 7357, Photogrammetry and Geomatics Group, National Institute of Applied Sciences (INSA Strasbourg), 24, Boulevard de la Victoire, 67084 Strasbourg, France

**Keywords:** Azure Kinect, RGB-D, TLS, MLS, 3D indoor reconstruction

## Abstract

With the development of 3D vision techniques, RGB-D cameras are increasingly used to allow easier and cheaper access to the third dimension. In this paper, we focus on testing the potential of the Kinect Azure RGB-D camera in the 3D reconstruction of indoor scenes. First, a series of investigations of the hardware was performed to evaluate its accuracy and precision. The results show that the measurements made with the Azure could be exploited for close-range survey applications. Second, we performed a methodological workflow for indoor reconstruction based on the Open3D framework, which was applied to two different indoor scenes. Based on the results, we can state that the quality of 3D reconstruction significantly depends on the architecture of the captured scene. This was supported by a comparison of the point cloud from the Kinect Azure with that from a terrestrial laser scanner and another from a mobile laser scanner. The results show that the average differences do not exceed 8 mm, which confirms that the Kinect Azure can be considered a 3D measurement system at least as reliable as a mobile laser scanner.

## 1. Introduction

The reconstruction of an as-built 3D model requires the acquisition of the current state of the building [1]. Point clouds, considered major inputs for 3D modeling of buildings, usually result from two main acquisition devices: terrestrial 3D scanners (TLS: terrestrial laser scanner) and dynamic 3D scanners (MLS: Mobile Laser scanner), which are based on the SLAM (simultaneous localization and mapping) technology.

Although it has the advantage of allowing the fast and accurate acquisition of a large volume of data, the approach based on the acquisition and segmentation of point clouds from TLS is expensive and not very user-friendly for indoor use and can be time consuming if a high level of detail is required. The use of SLAM technology can overcome this lack of convenience but at the expense of accuracy. Moreover, the prices of such systems remain inaccessible; whereas 3D reconstruction approaches that rely on vision only can also be used for this matter, they are often computationally intensive and suffer from a lack of robustness [2].

RGB-D cameras represent a low-cost alternative easy to use in an indoor environment. Interest in the use of RGB-D sensors and their applications in areas such as 3D reconstruction [3] and BIM completion [4] has grown significantly since the launch of Microsoft’s first Kinect sensor in 2010. The line of Kinect cameras has, ever since, known notable improvements with every updated version. The latest addition of the Azure Kinect differs from its predecessors in the fact that it supports four different depth sensing modes (wide field of view binned, narrow field of view binned, wide field of view unbinned, narrow field of view unbinned), and the color camera is of greater resolution (Microsoft, 2019). The precision (repeatability) of the Azure is also much better than the first Kinect versions [5].

Today, the evaluation of the potential of RGB-D sensors in BIM-oriented 3D reconstruction is a trending topic for the scientific community [3]. Indeed, the ability to successfully create complete and accurate models of indoor environments using a lightweight and inexpensive device such as the Azure Kinect would be highly advantageous for the BIM community.

Recently, several research studies have shown a real interest in the use of low-cost RGB-D systems for 3D modeling and BIM reconstruction. Some studies have focused on the evaluation of indoor and outdoor performance of these sensors. In fact, Ref. [5] propose a methodology to investigate the Kinect Azure sensor, evaluating its warm-up time, accuracy, precision, the effect of target reflectivity, and the multipath and flying pixels phenomenon. The results confirm the values reported by Microsoft. However, due to its acquisition technology, the device suffers from multipath interference, and its warm-up time remains relatively long (about 40–50 min) [5]. Ref. [6] solved the question about the Kinect Azure’s absolute accuracy by using a laser scanner as a benchmark, which extends the assessment to the whole field of view of the sensor instead of along the optical axis. Their results were consistent with the findings of [5]. The Azure Kinect shows an improvement over its predecessor in terms of random depth error as well as systematic error. This research, as well as others carried out about the investigation of various low cost RGB-D sensors [5,7], developed 3D modeling pipelines based on RGB-D data. Indeed, Ref. [3] recently proposed an automatic generation framework that transforms the 3D point cloud produced by a low-cost RGB-D sensor into an as-built BIM without any manual intervention. The plane extraction was based on the RANSAC (RANdom SAmple Consensus) algorithm enhanced by a new “add-drop” method. For semantic segmentation, a CRF (conditional random field) layer and a COB (convolutional oriented boundaries) layer were added at the end of the raw FCN (fully convolutional networks) architecture to refine the segmentation results. The experimental results show that the proposed method has competitive robustness, processing time, and accuracy compared with a workflow conducted by a TLS.

Other studies focus on combining data from low-cost RGB-D sensors with a TLS point cloud to complement it and improve its level of detail (LoD). Ref. [4] integrated the point clouds of small features (doors and windows) from the Kinect V2 sensor with a TLS model. The authors found that the segmentation accuracy for the Kinect data is highly dependent on the type of window frame or door jamb. Furthermore, the calibration of the two datasets remains mostly manual.

Some authors have been interested in improving RGB-D SLAM systems for the 3D reconstruction of buildings. Ref. [2] proposed a basic RGB-D mapping framework for generating dense 3D models of indoor environments. For image alignment, their approach introduced RGBDICP: an improved variant of the ICP (iterative closest point) algorithm jointly optimizing appearance and shape matching. The loop closure issue was addressed based on geometric constraints. RGB-D mapping also incorporates a “surfel” representation to handle occlusions. Although the global alignment process of RGB-D mapping is still limited, the system can successfully map indoor environments. The authors suggested that it would be useful to apply a visualization technique such as PMVS (patch-based multi-view stereo) to enrich the indoor model.

Due to the peculiar structure and scalability of indoor environments, the depth quality produced by RGB-D cameras and the SLAM algorithm are significant issues for existing RGB-D mapping systems [8]. Ref. [9] introduced an approach integrating SfM (structure from motion) and visual RGB-D SLAM to broaden the measurement range and improve the model details. To ensure the accuracy of RGB image poses, the authors introduced a refined false feature rejection modeling method for 3D scene reconstruction. Finally, a global optimization model was used to improve camera pose accuracy and reduce inconsistencies between depth images during stitching. This framework was reviewed by [8] in a comparative evaluation. The methodology proposed in [8] exploited all possible existing constraints between 3D features to refine the reconstructed model. The presented fully constrained RGB-D SLAM framework is centimeter accurate. Comparison of the proposed work with the visual RGB-D SLAM systems of [9] and SensorFusion demonstrated its usefulness and robustness.

The availability of RGB-D cameras to the public has provided the opportunity to develop several 3D reconstruction workflows. However, the challenges associated with this research area persist. It is necessary to distinguish between studies that deal with the modeling of individual objects and those that focus on the reconstruction of complete scenes. In the context of using RGBD sensors to model objects, the authors of [10] proposed an experimental method for the 3D reconstruction of a balustrade with Kinect V2. A circular network of frames from eight different perspectives was acquired around the object. As was previously suggested by the results of depth calibration, the sensor was placed at approximately 1 m of the object in order to minimize the global depth-related deformations. The accuracy of the resulting mesh was assessed with regards to a reference point cloud and mesh obtained by a measuring arm. While in both comparisons, the final error of about 2 mm on a significant part of the model is acceptable for a low-cost device such as the V2, the remaining deviations in the order of magnitude of one centimeter still represent a substantial percentage of the size of the object.

While research concerning the modeling of individual objects is widely available, studies related to the reconstruction of complete scenes remain limited. Constrained by the rather limited field of view of low-cost RGB-D cameras, the creation of a complete and reliable model of an indoor environment comes from several views acquired along a trajectory, each corresponding to a fragment of the scene. While this procedure often provides a global view of all surfaces, it suffers from significant odometry drift [11].

Frameworks developed in this sense can be classified into two categories: real-time online reconstruction (i.e., KinectFusion ([12,13]); BADSLAM [14]; ORB-SLAM2 [15]) and offline reconstruction (i.e., fully constrained RGB-D SLAM [8], visual RGB-D SLAM [9]), which requires post-processing. In both cases, several workflows have been developed and evaluated, and good accuracies have been achieved. However, the evaluation of the quality of the indoor scene models was performed either on synthetic scenes or through a visual evaluation of the results [11]). An actual evaluation with reference to a model of the same scene of supposedly better accuracy, obtained, for example, through a TLS, has not yet been performed, especially for the Azure Kinect.

The aim of this paper is to evaluate the potential of an RGB-D camera, precisely the Azure Kinect, for BIM-oriented indoor 3D reconstruction. The main contributions of our paper are threefold:

- An evaluation of the indoor performance of the Kinect Azure (Section 2.1).
- An evaluation of the quality of a 3D model reconstructed from this device (Section 2.2).
- A comparison with the clouds produced with both a terrestrial and a dynamic 3D scanner (Section 2.3).

The remainder of the present paper is organized as follows. Section 2 presents the methodology followed in order to investigate the performance and to assess the 3D reconstruction quality of the Kinect Azure. Section 3 treats the results of our study, which are further discussed in Section 4. Section 5 presents a conclusion and recommendations for future works.

## 2. Method

**Figure 1:**
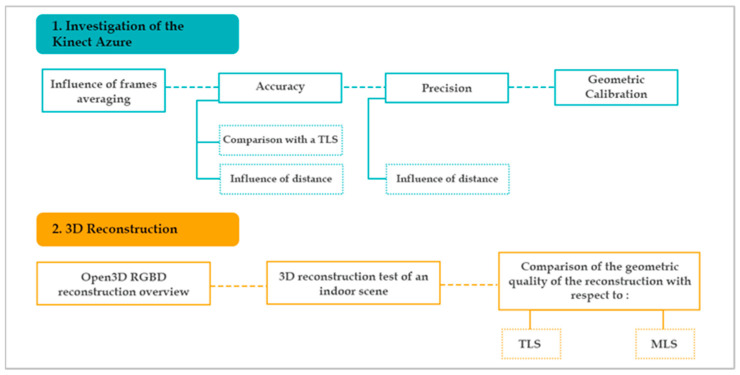
Overview of the general workflow.

The methodology followed in this work was composed of two main steps (Figure 1). First, we started with an investigation of the Kinect Azure by evaluating several parameters: the influence of the number of averaged images on the noise, the accuracy of the device referring to the measurements of a TLS, the influence of the distance on the accuracy, the precision as a function of the measurement distance, and, subsequently, a geometric calibration of the sensor. After analyzing the results of the investigation tests, we performed a methodological workflow test on Open3D in two different indoor scenes. Finally, we present a comparison of the geometric quality of the reconstruction provided with the RGB-D camera with respect to laser scanners, particularly a TLS and a MLS.

We note that other tests such as warm-up time and the influence of target reflectivity were also conducted but are not included in this paper as similar experiences were conducted by fellow researchers in [5] and yielded the same results.

### 2.1. Evaluation of the Kinect Azure

As reported in Figure 1, we conducted several experiments in order to assess the technical specifications as well as the performance of the camera with regards to several parameters. The adopted test’s methods are presented in the following subsections.

#### 2.1.1. Influence of Image Averaging

Image averaging assumes that the noise comes from a random source. Thus, the random fluctuations above and below the real image data gradually balance out as an increasing number of images are averaged. This technique is useful for reducing the inherent noise of the sensor and its technology existing in individual images [1]. 

**Figure 2:**
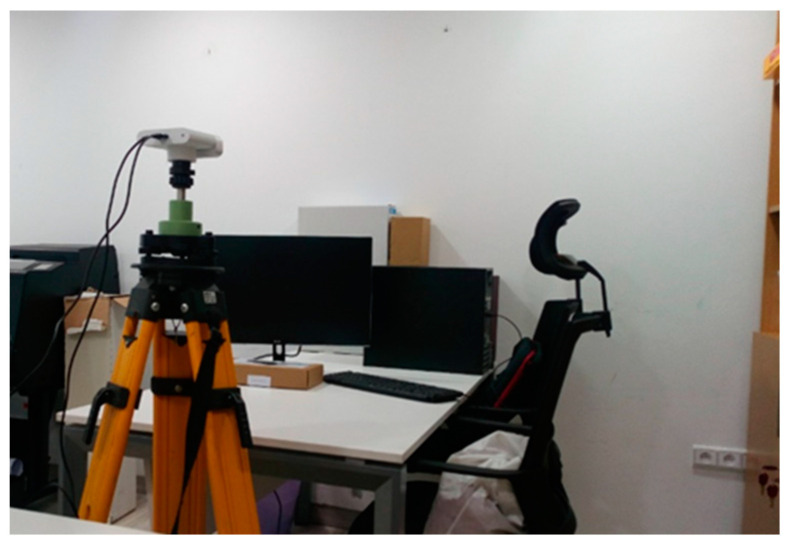
Indoor office scene used for testing image averaging.

Our experiment was performed in an office scene (Figure 2), where repeated measurements from a 900 mm range with respect to the wall were performed. This scene was chosen to represent a typical indoor environment, which is most often characterized by a set of occlusions. An analysis of noise variability as a function of the size of the averaged image set was performed with 20, 50, 100, and 200 successive depth maps. Using the acquired depth maps, for each pixel, the mean and standard deviation of the distance measurements were calculated.

#### 2.1.2. Accuracy

Accuracy reflects how close a measurement is to a reference value. To evaluate the accuracy of the Kinect Azure, we graphically represented the deviations in the measurements of our device compared with those from a TLS, which is supposed to be of higher accuracy. 

#### 2.1.3. Influence of Distance on Accuracy

**Figure 3:**
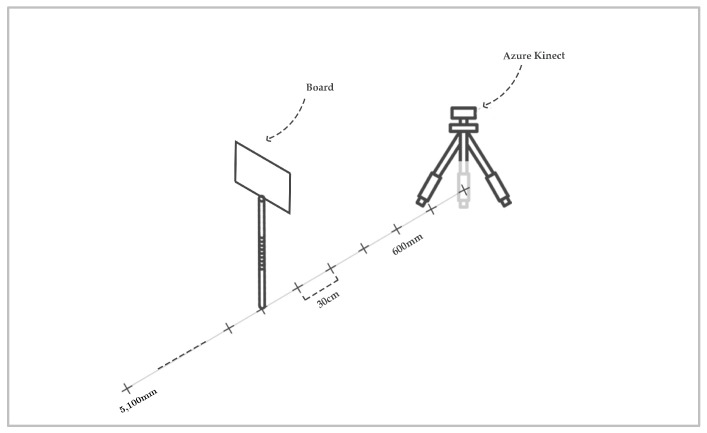
Operating modus for testing the influence of different ranges on measurement accuracy.

To study the influence of measurement distance on accuracy, we performed measurements from a static position of the Kinect by moving the target (whiteboard) along a perpendicular line to the camera plane and to distance marks stacked out by a total station (Figure 3). At each range, the position of the board was recorded by the total station to prevent it from changing orientation from one station to another. The differences between the distances from the Kinect (for each mode) and the total station were then calculated and analyzed.

#### 2.1.4. Precision

Precision reflects how reproducible measurements are. The data needed to evaluate the repeatability could be derived from the accuracy experiment. The standard deviations (Equation (1)) of the repeated measurements at each station and for all modes were calculated for each distance:

(1) $$\sigma =\sqrt{\frac{\sum_{i=1}^{n}{\left(x_{i}-\bar{x}\right)}^{2}}{n-1}},$$

where σ is the standard deviation, $x_{i}$ are the measurements, $\bar{x}$ is the estimated mean value, and n the number of observations.

#### 2.1.5. Geometric Calibration

One of the most widely used approaches for geometric calibration is that developed by [16]. It requires the camera to observe a planar target presented in a few different orientations.

**Figure 4:**
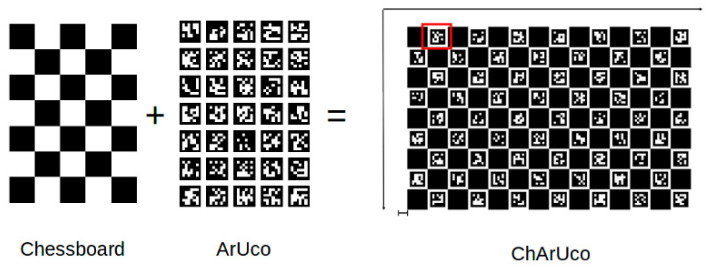
Design and parameters of the ChArUco board OpenCV.

In our case, the determination of the intrinsic parameters was performed separately for each camera onboard the Kinect Azure: a color camera and a depth camera. A board called ChArUco (Figure 4) printed on cardboard paper in order to minimize wrinkles was used. It is a flat board where uniquely encoded markers (ArUco) are placed inside the white tiles of a checkerboard. The corners of the markers can be easily detected in the images using the corner detection algorithms implemented in OpenCV (Open-Source Computer Vision Library), an open-source computer vision and machine learning software library. The checkerboard corners are then interpolated and refined. Finally, since the dimensions of the tiles were well known, we obtained correspondences between the 2D image coordinates and the 3D camera coordinates so that we could solve, in an iterative way, the camera model we established.

- Intrinsic parameters and distortions

**Figure 5:**
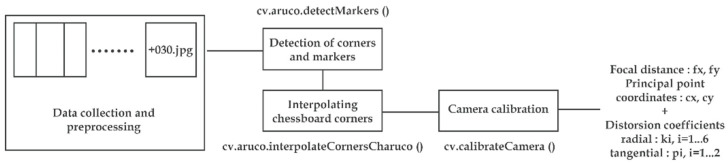
Workflow of geometric calibration on OpenCV.

From a static position of the camera, more than 30 images were taken, each time moving the target to occupy different distances and viewpoints, in order to properly reproduce the distortions created by the camera lenses. These images were processed using OpenCV to find the matrix of each camera, gathering the intrinsic parameters as well as the radial and tangential distortion parameters according to the Brown–Conrady model (Figure 5).

- Extrinsic parameters

This test consists of finding the parameters of the rotation–translation matrix that links the 3D landmarks of the two cameras. This requires, at least, a single image of the same target (the ChArUco board) taken by both cameras simultaneously. In our case, a set of five images was taken by moving the target to various positions. The parameters were then determined using a function available in the SDK (Software Development Kit).

### 2.2. 3D Reconstruction of Indoor Scenes

**Figure 6:**
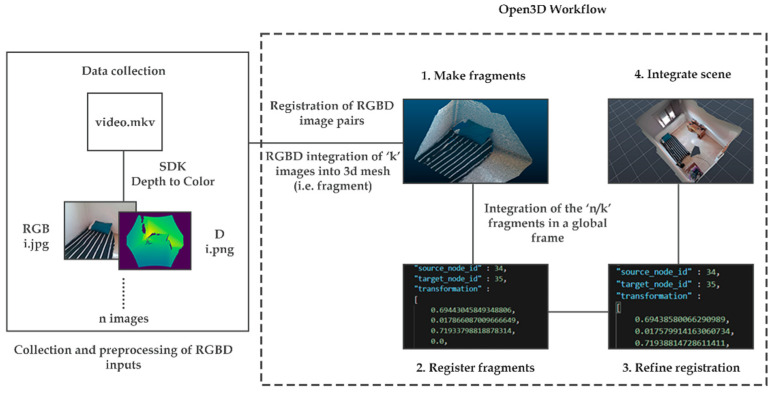
Diagram of the reconstruction of a 3D point clouds from RGB-D data on Open3D.

Open3D is a pipeline dedicated to 3D scene reconstruction from RGB-D data. It is based on the work of [11] and improved by the ideas of [17]. This framework was chosen for being an open-source project and for containing a set of carefully selected data structures and algorithms designed for manipulating 3D data. A diagram presenting the data preparation step and the processing part is shown in Figure 6.

The first step consists of collecting and preprocessing the RGB-D inputs. We used a custom C++ program, built using functions from the SDK, to extract and transform the color and depth images into a single coordinate system. The RGB-D input was then integrated into the open3D pipeline to generate a 3D mesh of the scene.

The experiment presented in this section had a double objective. The first one was to evaluate the performance of the Kinect Azure camera in a real indoor environment, while the second one was to evaluate the robustness of our methodological workflow in two scenes with distinct characteristics. The first scene was a furnished room with a single window. The second scene was a vacant office with several windows. 

Two depth modes WFOV (wide field-of-view) and NFOV (narrow field-of-view) unbinned were studied, in combination with the 1536p resolution of the color camera, which has a large field of view especially in the vertical direction. Furthermore, the quality of the reconstruction was assessed both by keeping the windows uncovered and then by covering them to block sunlight.

### 2.3. Quality Assessment of the Reconstruction with Respect to a TLS and a MLS

In this section, we conduct a comparison of the geometric quality of the 3D scene reconstruction resulting from the Kinect Azure and 3D models obtained by a TLS (FARO FOCUS S + 150) and a MLS (GeoSLAM ZEB Horizon). A comparison of the technical specifications of the three devices is given in Table 1. The comparison with the GeoSLAM is interesting since the acquisition principle as well as the registration process are quite similar to the Azure Kinect. It requires the user to continuously move through the room while scanning and performs a rough registration in real time through SLAM algorithms.

Before calculating the distances between the clouds, we first needed to align them. We chose the cloud from the TLS as the reference one since it is the most accurate. Then, the other clouds were aligned manually first, and then fine-tuning was performed using an ICP algorithm integrated in Cloud Compare. We use an RMS of 1.0 × 10^−5^, which consisted of the minimum improvement between two consecutive iterations to validate the registration result.

**Figure 7:**
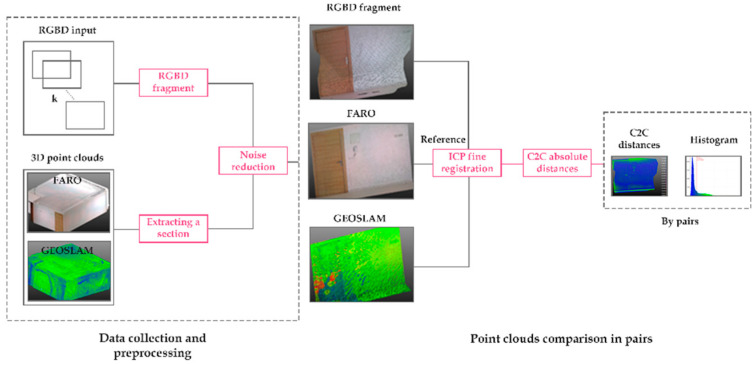
Workflow of data acquisition and comparison of point clouds two by two based on absolute distances C2C.

The comparison is based on absolute cloud-to-cloud (C2C) distances. This distance was chosen to facilitate the interpretation of the results. For each point of the compared cloud, the distance, in absolute value, of the nearest neighbor belonging to the reference cloud was calculated (Figure 7).

## 3. Results

In this section, we present the results of the performance tests as well as the 3D reconstruction. The conclusions drawn from the first tests allow a better understanding of the behavior of the device and confirmed its potential in terms of 3D reconstruction.

### 3.1. Azure Kinect Performance Tests

This first section presents the results of the experiments that focused on the technical specifications and the performance of the Azure Kinect camera.

#### 3.1.1. Influence of Image Averaging

**Figure 8:**
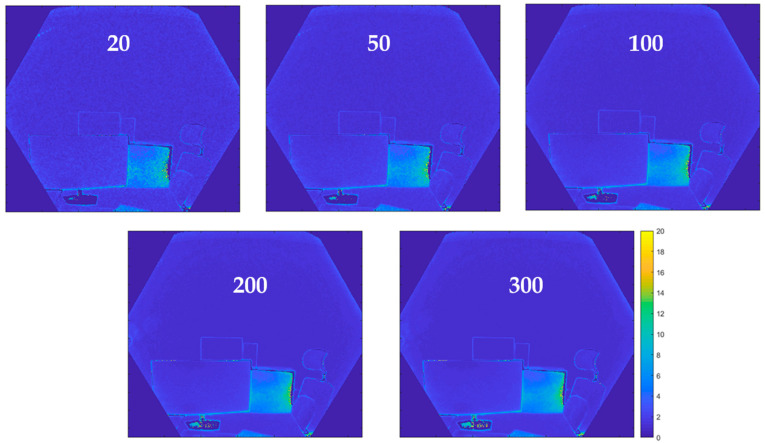
Evolution of standard deviations [in mm] of measurements according to the number of averaged frames.

Figure 8 presents the results obtained by calculating the standard deviations of the depth measurements and varying the number of consecutive frames. We can see that the standard deviations become higher on the edges of the objects as well as on the reflective surfaces (here, the metal plate). This effect is observed on all depth maps and does not necessarily disappear with increasing the number of frames. Furthermore, the visual rendering of the results becomes increasingly smooth by increasing the number of successive frames averaged (Figure 8). This aspect is particularly noticeable when going from 20 to 100 depth maps.

**Figure 9:**
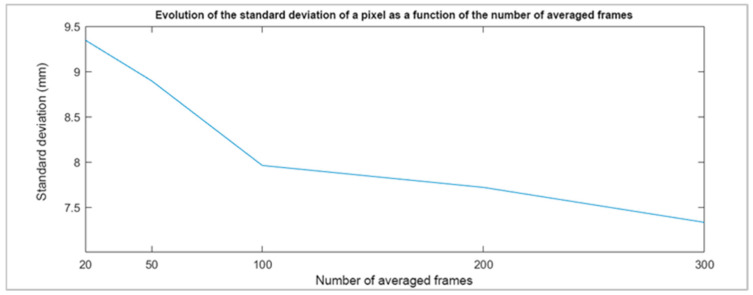
Evolution of the standard deviation of a pixel as a function of the number of averaged frames.

In general, the standard deviations of any pixel (not belonging to the edges of the objects) decrease by about 2 mm (from approx. 9.3 mm to 7.3 mm) when increasing the number of averaged frames from 20 to 300 (Figure 9). 

By going from 20 to 100 depth maps, the standard deviation decreases by 1.3 mm (from approx. 9.3 mm to 8.0 mm), which corresponds to 80% of the expected improvement with 300 frames. At this stage, adding more frames does not bring any significant improvement in the standard deviations. We conclude, therefore, that it is recommended to average 100 successive frames.

#### 3.1.2. Accuracy

**Figure 10:**
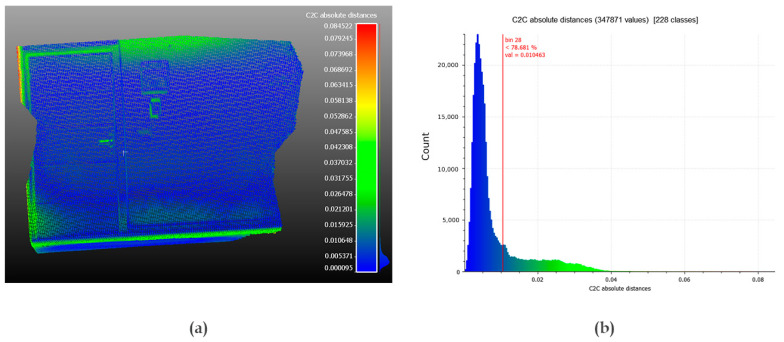
Comparison of the point cloud extracted from the Kinect Azure to the cloud from the TLS: (**a**) the differences between the two clouds [in m], (**b**) histogram of the values [in m].

A point cloud of the same indoor scene was extracted from the Kinect Azure and compared with the point cloud from a 3D scan performed by the TLS. The average distance calculated between the two clouds reaches 8 mm, while the standard deviation is 8 mm (Figure 10). The histogram of the distances shows that most of the deviations are less than 10 mm, which is acceptable for standard topographic surveys. It is important to note that the most pronounced gaps are located on the contours of the details, including walls, doors, electrical box, and at the contours of the intercom. A more detailed demonstration of the procedure, as well as an in-depth analysis of this comparison, will be addressed in a later test.

#### 3.1.3. Influence of Distance on Accuracy

**Figure 11:**
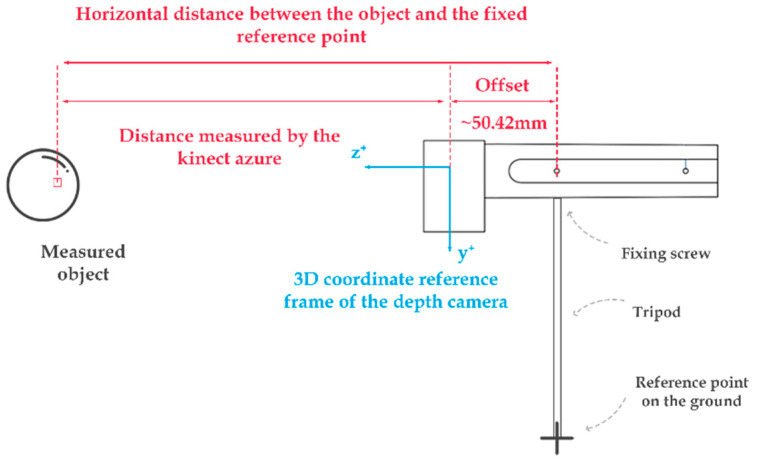
Schema of the difference between the distance measured by the Kinect and the true horizontal distance from a point on the ground.

First, we notice that there is an offset between the camera mounting screw and the depth camera lens corresponding to the origin of the camera coordinate system. Referring to the data sheet of the sensor, this offset is estimated at 50.4 mm (Figure 11). This value was taken into consideration to correct measurements. Using its mounting screw, the camera was accurately placed on a level topographic base and mounted on a tripod.

**Figure 12:**
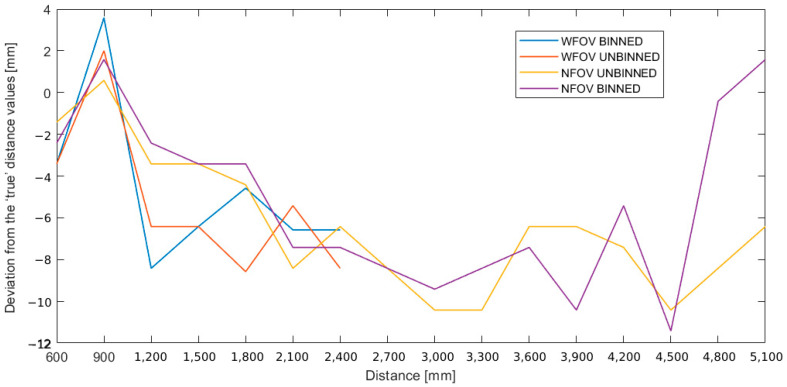
Evolution of the deviations, in mm, from the “true” distances.

We can notice that there is no direct and clear correlation between the evolution of the accuracy and the distance of the measurements. However, we can say that the accuracy of our device tends, generally, to decline with increasing distance, ranging from +4 mm to −11 mm (Figure 12). This is not the case for large distances where the deviation seems to oscillate around the same value. The graphs corresponding to the different modes supported here show almost the same pattern with differences in values. In general, the deviations do not exceed −11 mm except for the NFOV binned mode at 4500 mm. For small distances, the two variants of the narrow mode seem to give the smallest deviations compared with those of the wide mode.

#### 3.1.4. Influence of the Distance on Precision

**Figure 13:**
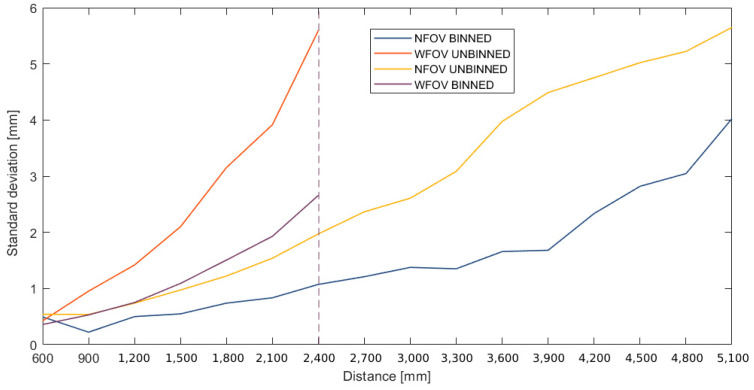
Evolution of standard deviations of measurements as a function of distance [in mm].

In general, the standard deviations for all modes tend to increase with distance but do not exceed 6 mm (Figure 13), which is interesting for close-range applications. The narrow-mode variants show better results than the wide mode. Similarly, for the same mode, the binned variants show smaller deviations compared with the unbinned.

In summary, by examining the effect of frame averaging on the noise, it can be concluded that the averaging of 100 successive maps is sufficient and gives the best results (1.3 mm decrease in the standard deviation going from 20 to 100 depth maps). We also examined the accuracy of our instrument by comparing it with a TLS. The mapping of the differences between the two point clouds showed that they do not exceed 8 mm. Such accuracy motivates the use of the Azure Kinect for accurate indoor mapping. The analysis of the variations in the accuracy and precision as a function of the distance show that the narrow-mode variants present the best results. The wide mode, on the other hand, has the advantage of allowing a much wider shot. We conclude, therefore, that the choice of mode should be made following the aspects that interest the user. Our results confirm that the standard deviation of the Kinect Azure does not exceed 6 mm at a maximum range of 5100 mm.

### 3.2. 3D Reconstruction

Before presenting the result of the 3D reconstruction, it is interesting to assess the time required for applying the workflow, from data acquisition to the final result, for the two scenes (Table 2).

The long processing time, in the case of the room, is due to the high number of frames, which result from the use of an FPS (frames-per-second) rate of 30, in addition to scanning the scene twice due to the use of the NFOV mode, which has a limited field of view in the vertical direction.

**Figure 14:**
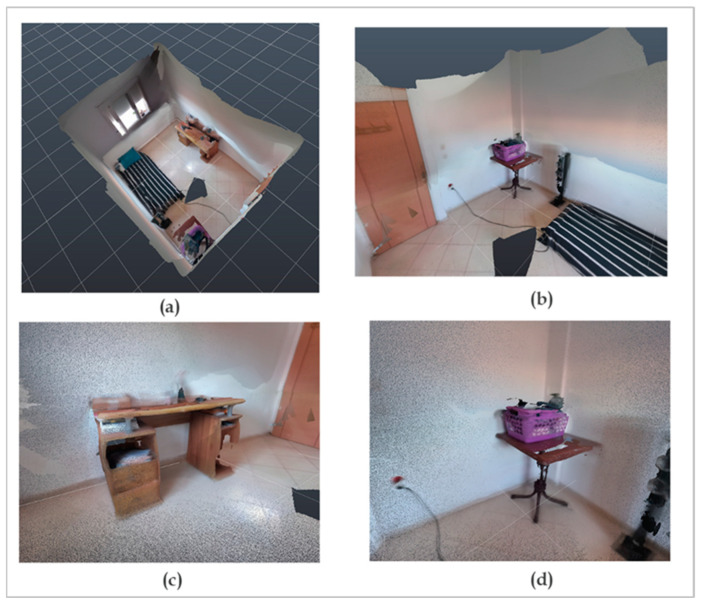
Result of the indoor 3D reconstruction of the room: (**a**) global view of the room from the top; (**b**–**d**) focus on corners and objects.

**Figure 15:**
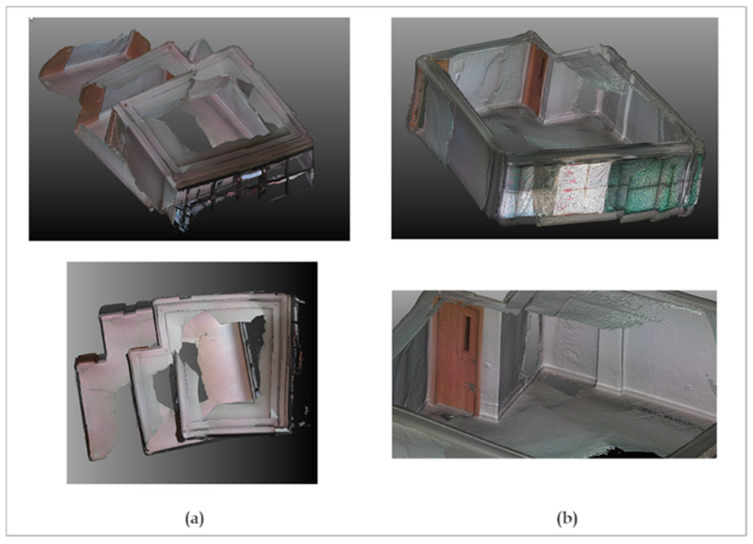
Results of indoor 3D reconstruction of the office scene. (**a**) by keeping the windows uncovered; (**b**) by covering the windows.

The results of the reconstruction of the two scenes are shown in Figure 14 and Figure 15.

### 3.2.1. Evaluation of the Quality of the 3D Reconstruction

Concerning the first scene (the room), in the absence of a reference model, we opted for a visual evaluation. The reconstruction performed can be qualified as successful. The loop closure, which means that a correspondence was made between the details captured at the beginning and the same details were captured at the end of the acquisition, was performed correctly, and odometry drift was not observed, even with the presence of an uncovered window. The result of the color integration was also visually correct (Figure 14).

Although the NFOV modes enable a better depth range, they are not suitable for capturing large scenes in an efficient way because their vertical field of view is rather limited, which does not capture the full height of the walls, even when scanning from the maximum range. On the contrary, the WFOV modes have a wider field of view (horizontally and vertically), which allows for reducing the number of frames. It can be noticed that the depth range offered by the WFOV mode is sufficient for a close-range modeling application.

It is important to note that the occlusion problem persists (Figure 14c,d). Due to the nonvisibility of objects from all angles, there is a lack of depth data for some objects. Indeed, in order to avoid problems during the registration part of the post-processing, we cannot dwell too much on all the details of the scene. It is, therefore, judicious to try to simplify the trajectory of the camera so that the solution on Open3D converges correctly.

**Figure 16:**
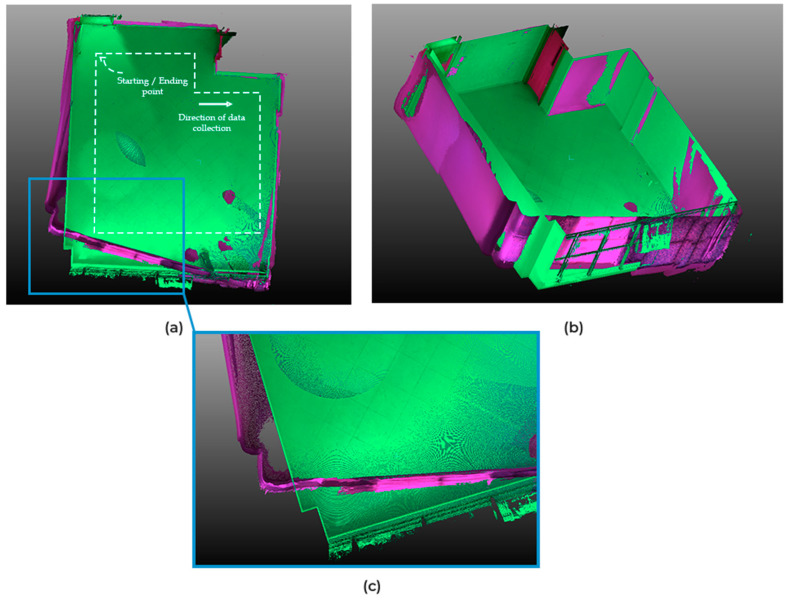
Odometry drift effect highlighted by superposing point clouds from TLS (green) and point clouds from Kinect Azure (purple). (**a**) Top view of the point clouds, indicating the start point and the trajectory. (**b**) Perspective view. (**c**) Zoom on a corner of the room to highlight the odometry drift.

Concerning the office scene, a first attempt was made by keeping the windows exposed. As expected, the presence of shiny surfaces led to erroneous depth measurements. This induced an incorrect reconstruction of the scene (Figure 15a). We observe that the loop closure failed, as indicated by a staircase effect when the fragments were realigned. To confirm this, a second attempt was made by covering the window with curtains (Figure 15b). At first glance, the loop closure appeared successful, and the fragments were aligned correctly. However, an odometry drift effect persisted. This was highlighted by overlaying this result with the point cloud from the TLS (Figure 16). We note that this effect began, roughly, halfway through the windows and continued to accumulate as we went along. Although loop closure in this case mitigated these errors, it was apparent that the reconstruction deviated significantly from reality at several locations in the scene, especially when we got close to the windows.

We should note that in both cases, the acquisition was tested with both modes, NFOV and WFOV, and that this did not significantly affect the final result. The use of the WFOV mode allowed capturing the whole scene in a single turn by holding the camera regularly in front of the surface to be scanned. However, corners and wall intersections should be scanned carefully since they very often resulted in zero-value pixels due to the multipath effect. Averaging several successive depth maps, in the form of fragments, helps to remedy this problem somewhat. However, this still needs to be taken into consideration when scanning the scene.

In conclusion, the performed experiments are useful for identifying, on the one hand, the potential of the sensor in 3D scene reconstruction, and on the other hand, its shortcomings and limitations in order to propose possible improvement paths. Indeed, the combination of the WFOV mode with a high resolution of the color camera allows efficiency in terms of data acquisition time (by scanning the whole scene in a single turn), file size in storage, and processing time. However, it was found that scenes containing a large area of windows can be problematic due to erroneous depth values. This is not the case for small windows found in typical indoor environments, which do not seem to affect the final result since the resulting zero-pixel values constitute only a small part of the captured depth maps.

### 3.2.2. Comparison of the Geometric Quality of Reconstruction (TLS–MLS)

**Figure 17:**
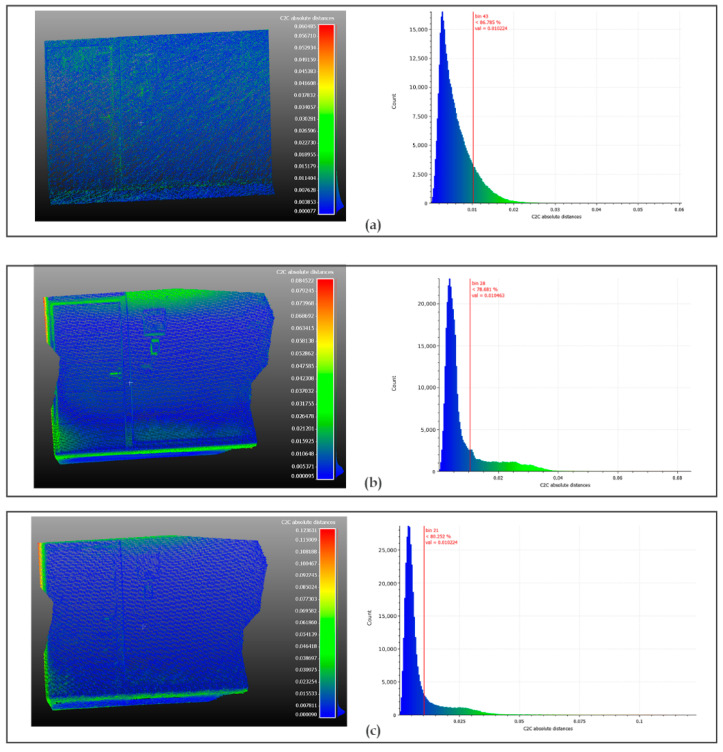
Means and standard deviations of C2C distances between the point clouds: (**a**) TLS vs. MLS, (**b**) TLS vs. Kinect Azure, (**c**) MLS vs. Kinect Azure.

The result of comparing the point clouds is presented in Figure 17. Table 3 summarizes the means and standard deviations observed by performing the following comparisons of point clouds: (1) the TLS with the MLS; (2) the TLS with the Kinect Azure, and (3) the MLS with the Kinect Azure. The mean differences between the clouds are less than 10 mm, which is very satisfying.

Visually, the 3D mesh obtained from the Kinect is smoothed at the corners as well as at the edges of the walls. This effect explains a large part of the deviations compared with the other two clouds. The algorithm used encountered more difficulties in matching the images of the second scene (office) than those of the first scene (room). This can be explained by the fact that the room contains distinctive points that facilitate the task of image matching, unlike the office, which has only white walls. It would have been useful to put, for example, colored stickers on the office walls.

For each of the two clouds, we extracted a part of the wall and interpolated a corresponding least squares plane. Then, the deviations—in absolute values—were calculated for these planes and reported in Table 4. The TLS point cloud presents a standard deviation of 0.7 mm, while the MLS point cloud presents a deviation of 3.7 mm from the adjusted plane. This confirms that the deviations between the clouds from the two devices are due to the noise inherent to the measurements made with the MLS.

The differences in the point clouds resulting from the two scanners with the Kinect Azure are almost the same (about 8 mm). We also note that the distributions are left skewed (Figure 17), which means that most of the deviations are less than 10 mm (78% of the values for the TLS and 86% of the values for the MLS). However, these values alone are not sufficient to validate the quality of the geometric reconstruction.

**Figure 18:**
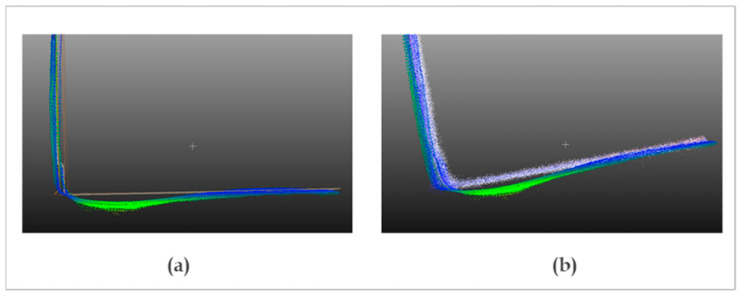
Comparison of the intersection of the wall with the ground. (**a**) TLS (white) vs. Kinect Azure (color), (**b**) MLS (white) vs. Kinect Azure (color).

Obviously, the deviations are higher in the salient elements located on the wall plane (electrical box, intercom, wrist), in addition to the edges of the wall and the door. Deviations are also observed in the intersections of the wall with the ground as shown in Figure 18.

The error on the edges of the details is probably due to the angle of the shot. Unfortunately, it is not practical to capture objects from all angles, especially small ones. This is the case for the box, the handle, and the intercom.

**Figure 19:**
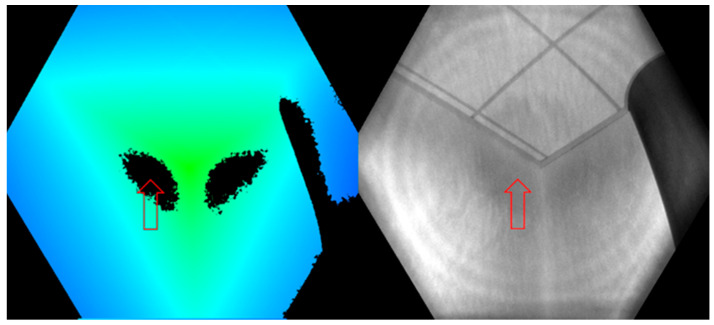
Multipath phenomenon observed on the intersection of two walls at the upper section corner of a room, as indicated by the red arrows. Pixels in black have zero depth values: depth image (**left**), passive infrared image (**right**). Microsoft.

The error that affects the intersections is suspected to be from the multipath phenomenon. Indeed, these parts are often not captured at once by the depth sensor because of the angle of incidence of the infrared signal. The signal bounces off several surfaces, for example between the ends of the wall and the floor, which results in pixels of zero values (Figure 19). This is one of the main limitations of using this type of sensor for indoor reconstruction: Even if a detail of the scene is visible, depth information may not be available for the entire frame in question. This effect can be alleviated by varying the angle of view and by taking several successive images, but a right ratio between acquisition time and required level of detail must be defined in future works. Another potential solution would be to perform a depth calibration.

## 4. Discussion

The indirect ToF technology has proven its robustness in several applications, including indoor 3D reconstruction. The new version of the Kinect sensors brings even more improvements, mainly centimeter-level precision and accuracy and the introduction of several depth modes—each tailored to a different scenario—in addition to the integration of an inertial measurement unit.

As with the second Kinect version, the Azure camera needs to be warmed up for a relatively long time in order to stabilize the output. The order of magnitude of the improvement due to a 40–50 min warm-up is approximately 2 mm. 

Overall, the performance investigation tests yielded interesting values with regard to accurate indoor modeling applications. The methodological workflow developed for 3D reconstruction was carried out using the open-source framework Open3D. It is particularly interesting for customizing the processing chain, allowing the implementation of algorithms, which was the case for this study.

It is also worth mentioning that the effect of distortion in the intersections of surfaces (between walls and the floor, for example). This effect persists even after performing geometric calibration to correct for distortion effects caused by the camera lens. Probable causes have been discussed, such as the multipath phenomenon or the lack of depth calibration, which requires further testing in other environments. 

In conclusion, the Kinect Azure has several advantages for 3D indoor reconstruction. Its small weight and miniaturization make it easy to handle. The large number of frames per second is useful for avoiding the blur effect when scanning the scene. Finally, its cost is exceptionally low compared with professional RGB-D sensors.

The negative points that we have highlighted concern the data acquisition solution available with the SDK. For instance, the SDK does not present real-time feedback for visualizing the output of the camera as we go through the scene. The need to connect two cables, one for the power supply and the other for the connection with the workstation, affects practicality since the device must remain connected to a power source to work. It would have been better to combine the two into one cable so that the device can be powered directly from the laptop. Finally, the camera also performs poorly with highly reflective materials, which are quite common in indoor scenes. Therefore, particular care must be taken during data acquisition.

## 5. Conclusions

The Azure camera is the latest addition to Microsoft’s line of Kinect sensors. According to the manufacturer, it has notable performance improvements over its predecessors. In this paper, we performed a series of experiments to evaluate the potential of the new Kinect in the context of 3D indoor reconstruction. The aim of this paper was to draw first conclusions about this device and to see if it could potentially become a low-cost alternative to close-range laser scanners for 3D enthusiasts. The main contributions of our work are threefold: (a) the evaluation of the indoor performance of the Azure, (b) the construction of a 3D model of real indoor scenes based on Kinect acquisition, and (c) the evaluation of its geometric quality, referring to a more accurate reference as well as the comparison of the resulting model with those issued from both a terrestrial and a mobile 3D scanner.

Based on our experiments, we can say that in terms of accuracy and precision, this sensor has a significant potential in 3D indoor modeling applications. It offers a variety of modes that users can adapt to their needs, as well as competitive resolutions compared with other low-cost sensors. However, the sensor still has some drawbacks, including the long warm-up time. As with many other sensors based on ToF technology, the Azure is also affected by the phenomenon of flying pixels and multipath interference. The 3D reconstruction scene confirmed the initial conclusions concerning the potential of the Azure in BIM applications. Nevertheless, it seems that the robustness of the solution depends, to a large extent, on the architecture of the scene in question. This conclusion, however, needs to be investigated further by experimenting with other indoor scene configurations. A comparison of the point cloud from the Azure with that from a TLS and another from a MLS showed that the average differences between the measurements from the laser scanners and the measurements from the Azure Kinect do not exceed 8 mm.

In conclusion, the Azure Kinect is a promising device. It surely has potential that deserves to be exploited for a wide range of applications, mainly 3D interior reconstruction and as-built BIM.

In the future, some improvements that we suggest concern the development of a data acquisition application with a better interface for visualizing the data stream during scanning. It is one of the challenging aspects for SLAM sensors that requires achieving complex calculations and displaying large amounts of data simultaneously and in real time. This could be partially overcome by mapping the computed trajectory of the sensor. Other interesting perspectives would be implementing the integrated IMU (inertial measurement unit) in the reconstruction workflow to mitigate the effect of odometry drift, extending the experimentation to different environments such as adjoining rooms, and exploring the contribution of depth calibration. Although we compared the computed Azure Kinect point cloud with those produced by a TLS and an MLS, it should also be tested with segmentation algorithms that integrate into the scan to BIM workflows. 

## Figures and Tables

**Table 1:** Comparison of the technical specifications of three devices (Microsoft Kinect Azure 2019, FARO 2019, GeoSLAM 2019).

	FARO Focus S Plus 150	GeoSLAM ZEB Horizon	Kinect Azure NFOVU
Range (m)	150	100	3.86
FOV (degrees)	360 × 300	360 × 270	C: 90 × 74.3
D: 75 * 65			
Weight (g)	4200	3700	440
Scanning velocity (pts/s)	2,000,000	300,000	-
Relative precision (mm)	1	10–30	11
Raw data file size (MB/min)	40–50	100–200	2000–3000

**Table 2:** Time spent during data acquisition and processing for 3D reconstruction.

Phase	Operation	Processing Time	
		Room	Office
Data acquisition	Scanning with the Kinect	2 min 13 s	2 min 05 s
Preprocessing	Image extraction and alignment on the same coordinate system	27 min 34 s	22 min 46 s
3D reconstruction	Fragment creation	3 h 33 min 26 s	2 h 31 min 25 s
	Fragment registration	1 min 34 s	2 min 24 s
	Fine registration	20 s	45 s
	Integration	11 min 11 s	42 min 09 s
	Total	4 h 16 min 18 s	3 h 41 min 34 s

**Table 3:** Mean C2C distance [in mm] and standard deviations [in mm] between point clouds.

	Average Distance (mm)	Standard Deviation (mm)
TLS–MLS	6	4
TLS–Kinect Azure	8	8
MLS–Kinect Azure	8	10

**Table 4:** Average values (in mm) and standard deviations (in mm) of the deviations between the clouds—from TLS and MLS—from the interpolated planes.

	Deviations to the Interpolated Plane	
	Average Deviation (mm)	Standard Deviation (mm)
TLS	0.7	0.7
MLS	4.6	3.7

## Data Availability

Data supporting reported results can be found at the following link: https://mega.nz/folder/ctIFRSyL#p80SkY5t_BBoDTSm_9WsqA (accessed on 15 November 2022).
